# Development, preparation, and evaluation of a novel dotted lateral flow immunochromatographic kit for rapid diagnosis of dermatophytosis

**DOI:** 10.1038/s41598-023-27443-4

**Published:** 2023-01-05

**Authors:** Hassan Aboul-Ella, Rafik Hamed Sayed, Heidy Shawky Abo-Elyazeed

**Affiliations:** 1 Department of Microbiology, Faculty of Veterinary Medicine, Cairo University, Giza, Egypt; 2 Department of Microbiology, Central Laboratory for Evaluation of Veterinary Biologics (CLEVB), Agricultural Research Center (ARC), Cairo, Egypt

**Keywords:** Immunology, Microbiology

## Abstract

Dermatophytosis is a widely spread contagious zoonotic disease, affecting both man (tinea) and animals (ringworm). This disease is caused by a group of closely related keratinophilic fungi known collectively as the dermatophytes group. Although the wide distribution of dermatophytosis cases throughout the whole world and its adverse clinical effect on human health, economical effect on productive animals, and pet animal welfare, there is no rapid accurate diagnostic tool for such disease. The current conducted study tries to accomplish the difficult equation by achieving an accurate, sensitive, specific, user-friendly, rapid, robust, device-less, deliverable to end-users, and economic cost for the development and production of diagnostic kits. Through the development of a rapid diagnostic kit based on immunochromatographic assay with three major affordable reproducible production stages; preliminary stage, developmental and standardization stage, and evaluation stage. Obtaining dermatophytes-specific polyclonal antibodies against criteria-based selected dermatophytes strains associating proper gold nanoparticle preparation, characterization, and conjugation, with proper loading of the different bio-reactants on the efficiently laminated and fabricated lateral flow strips were the main challenge and control points through the whole process. Also, as a result of examining 100 animal samples using the new kit, the κ coefficients of the kit with the direct microscopy while the kit with the culture were 0.44 and 0.76, respectively. Therefore, the newly designated and developed kit showed a very promising competitive diagnostic result within 5–7 min through easy-to-be-performed three steps.

## Introduction

Dermatophytosis is a zoonotic disease caused by a closely related group of keratinophilic hyaline fungal agents called the dermatophytes group. It includes three main genera and more than fifty species^1^.

Dermatophytosis is a highly contagious and widely spread disease that infects both man and animals with increased and regular incidence all over the year throughout the whole world^2^.

Despite the vital need of obtaining a precise diagnosis of dermatomycoses cases, scientific funding and diagnostics investment in applied medical mycology have lagged. Public and private investment in therapeutic interventions, on the other hand, is constantly increasing. In the United Kingdom (UK), for example, mycological diagnostic research received about 1.9% to 2.8% of the funding awarded to more than 6125 studies in infectious diseases conducted between 1997 and 2020, while clinical research in the same medical field received only 12.7% of this allocation, with overall 14–15% mycological research funding^3^. The fraction of this small proportion of funding which included diagnostics studies in this published paper is likely to be less than a fifth of the 12.7 percent, suggesting that research in the UK, as an example of a developed country, fungal diagnostics research may receive less than 0.1% of government research funding, and even less in developing countries. This appears to be an insufficient investment in a significant medical need^4^.

Up to date, dermatophytosis diagnosis is limited to direct, rapid with low sensitivity and specificity several wet mounts techniques, sophisticated with highly skilled personnel requirement molecular assays, and gold standard with long time consumption conventional culturing and biochemical testing. All of the previously mentioned existing diagnostic methods are lab-confined techniques with no availability of field, handheld, or bedside tests. The different aspects of difficulties facing dermatophytosis diagnosis lead to mistaken diagnoses and faulty prescriptions of antifungals with their expected long list of side effects on man and animals^5^.

Direct potassium hydroxide (KOH) microscopy and fungal culture are considered the gold standard for diagnosing dermatomycosis although other molecular diagnostic techniques are existing. Direct KOH microscopic examination and other available direct microscopy-based techniques appear simple but require skill for appropriate sampling, resolving the collected skin scrapings and nail pieces, and identifying the organisms^5^.

Although all culture-based techniques have high specificity, they share costly procedures, a long time to result, and intensive lab work^6–11^.

Also, molecular-based diagnostic technology to amplify fungal nucleic acid with a prominent remarkable specificity and sensitivity has become a promising standard diagnostic method for laboratory diagnosis of dermatophytosis^12^. However, it is heavily confined to a laboratory facility, and experienced staff which so, unfortunately, are not available and reachable to most public health communities and remote countryside as well as low-income countries^13^.

As well as, it is worth to be mentioned that there were few previous trials to incorporate antibody-based immunochromatographic assays in the field of dermatophytosis diagnosis but with different approaches than those performed and obtained through the current study^14–18^. All those trials shared an expensive and sophisticated hard to be-established in developing countries monoclonal antibody production technique, limited nature of samples that the kit is able to deal with, mainly nails, and/or the usage of the dermatophyte allergen that is not commercially available today on the level of the preparation of the used antigens. On the other side, the current work covered all those unclearly identified stages of development and production, highlighting the economic impact of using polyclonal antibodies (pAbs) instead of monoclonal antibodies (mAbs) which will lead to easy and widespread application of the developed kit. Also, the current work represents a leading implementation of the dermatophytes lateral-flow diagnostic kit in veterinary practices. The currently developed kit in the form of packaged ready to be used based on laboratory production circumstances is 7 United states dollars which is a very competitive price in comparison with other available commercially produced kits in Japan which cost 2000 yen (about 14.5 United States dollars) and in Europe which costs 20 euro (about 21 United States dollars).

With the wide impediments in the field of diagnostic mycology especially in low-income countries as the lack of specialized clinical mycologists and low-cost, rapid, sensitive as well as specific diagnosis requirements, the need for a competitive diagnostic technique with sensitive, specific, and fast results obtaining and interpretation is considered a medical priority in the field of dermatophytosis diagnosis^4,19^.

The current conducted study aimed to empower the field of diagnostic mycology in general and dermatophytosis-related cutaneous infection specifically, by a rapid, easy-to-be-performed, and economically to be developed, manufactured, and available for clinicians, and animal caregivers all over the world.

## Results and discussion

### Pre-kit development stage (preliminary stage)

#### Agar gel precipitation/Ouchterlony’s test (AGPT)

During the immunization protocol, serum was tested against the prepared antigens by AGPT, it showed no results after the 3rd injection, a weak result after the 5th injection while strong clearly defined results started after the 8th injection. After the purification of pAbs, purified pAbs were tested against the prepared antigens, AGPT was still showing strong results as a confirmatory step for the success of the pAbs separation and purification with caprylic acid. Before the involvement of the purified pAbs in the LFIA preparation, the purified dermatophyte species-specific pAbs were tested against: The prepared different dermatophyte antigens for antigenic relationship determination, and no antigenic relationships were found between the different dermatophyte species prepared antigens, as well as other non-dermatophyte keratinophilic fungal antigens prepared from the following genera and species (*Candida albicans*, *Candida tropicalis*, *Candida glabrata*, *Candida krusei*, *Malassezia pachydermatis*, *Aspergillus niger*, *Aspergillus fumigatus*, *Aspergillus flavus*, *Fusarium oxysporum*, *Fusarium chlamydosporum*, *and Alternaria alternata*), also no antigenic relationships were found between dermatophyte antigens and other non-dermatophyte keratinophilic fungal antigens.

The specificity of the produced polyclonal antibodies was completely studied and covered using Ouchterlony’s test, which is mainly used to determine the antigenic relationships, through three different precipitation line formations: single line = no relationship, two crossed lines = complete relationship, or two spur forming lines = incomplete antigenic relationship.

In the case of the prepared species-specific anti-dermatophytes Abs, only single precipitation lines were obtained between each produced dermatophyte antigen and its specific antibodies (Specific anti-species polyclonal antibodies), Supplementary Fig. S1; (Supplementary Fig. A–D). When using a mixture of the produced antibodies (multi-species specific polyclonal antibodies), it reacted with all dermatophytes’ species-specific antigens, Supplementary Fig. S1; (Supplementary Fig. E). As well as a mixture of the produced antibodies (multi-species specific polyclonal antibodies) was tested against a wide list of previously mentioned non-dermatophytes keratinolytic fungal antigens, however, no precipitation lines were obtained representing no antigenic relationships, Supplementary Fig. S1 (Supplementary Fig. F).

### Kit development stage (developmental and standardization stage)

#### Preparation and conjugation of the purified rabbit polyclonal antibodies with the colloidal gold nanoparticles (Au NPs-pAbs coupled bio-conjugate)

Firstly, the obtained colloidal gold nanoparticles were confirmed to be 40 nm in diameter. As well as, the performance of any newly developed lateral immunochromatography-based kit mainly depends on a variety of parameters, such as the antibody labeling by the gold nanoparticles, the pH value set within the strip microenvironment, and at which the Ag–Ab immune-complex is expected to occur if the target analyte exists, amount of specific antibodies required for optimal immunological coupling, and finally the antigen/antibody loading concentration of the test/control spots. Based on the colloidal gold preparation methodologies, the most stable and unaggregated immune-gold bio-conjugate is achievable at a pH that is slightly higher or near the isoelectric point of the conjugated protein. The highest adsorption capacity of antibodies on the surface of the gold nanoparticles was achieved by fixed unchanged ruby red color formed in the pH series between 7.2 and 7.6. 0.02 M potassium carbonate (K2CO3) was used to adjust the pH of colloidal gold to 7.4. The amount of antibodies that can form stable conjugates and optimally reacted test and control spots is another key factor for the success of the newly developed kit. To determine the optimal amount of antibodies, different concentrations of anti-dermatophytes IgG were tested. However, Au NP solution (100 μl) containing 1.0 μg of pAbs of anti-dermatophytes IgG on the conjugate pad could remain as stable ruby red reactions after exposure to the positive and negative control solutions^20–22^.

#### Lateral flow used membranes illustration

The sample pad’s role is to receive the sample or the extracted sample antigens carried by the extraction solution, the conjugate pad is that pad where the conjugate is temporarily immobilized and represented on the lateral flow strips, the NC membrane function is to indicate the presence or absence of the target analytic in liquid samples. Several reasons contributed to making NC the preferred LF solid phase: High affinity of protein adsorption, the chemical properties that led to stable protein adsorption levels in both dry and wet conditions of the NC membrane, and nitrocellulose membranes can be cast that have pores sufficiently large to allow a lateral flow of fluid in a reasonable time, absorbent pad to offer a consistent absorbency for test reproducibility, also allowing quick clearing of the membrane by absorbing higher volumes of liquid and access to the read-out in a shorter time, and finally the PVC sheets that acting as a base upon which all previously mentioned membranes and pads are adhered and oriented.

#### The prepared and formulated extraction solution

Due to the solid nature of the dermatological affection samples (hair, skin scraps, scales, nails), a “pre-application sample processing step” must be performed to extract the dermatophytes’ antigenic structures from the collected sample. Distilled water was used as a carrier stage of the rest of the extraction solution components and at the same time as a dissolving agent, glycerin as a moisture-providing agent In direct contact with the sample surface which lead by its role to shorten the time required for antigen extraction, Potassium hydroxide 5% and hydrogen chloride 30% as a pH adjusting agents, Sodium dodecyl sulfate an anionic surfactant, Dimethyl sulfoxide a polar solvent for polar and non-polar substances, Propylene glycol as a non-ionic surfactant, organic solvent, as well as excellent grease and fat solvent, Diazolidinyl urea an antimicrobial preservative to increase the shelf life of the solution, and Ethylene-diamine-tetra-acetic-acid (EDTA) as an astringent for the cuticle and keratin layer.

#### Principle of the developed LFIA strip

**Figure 1:**
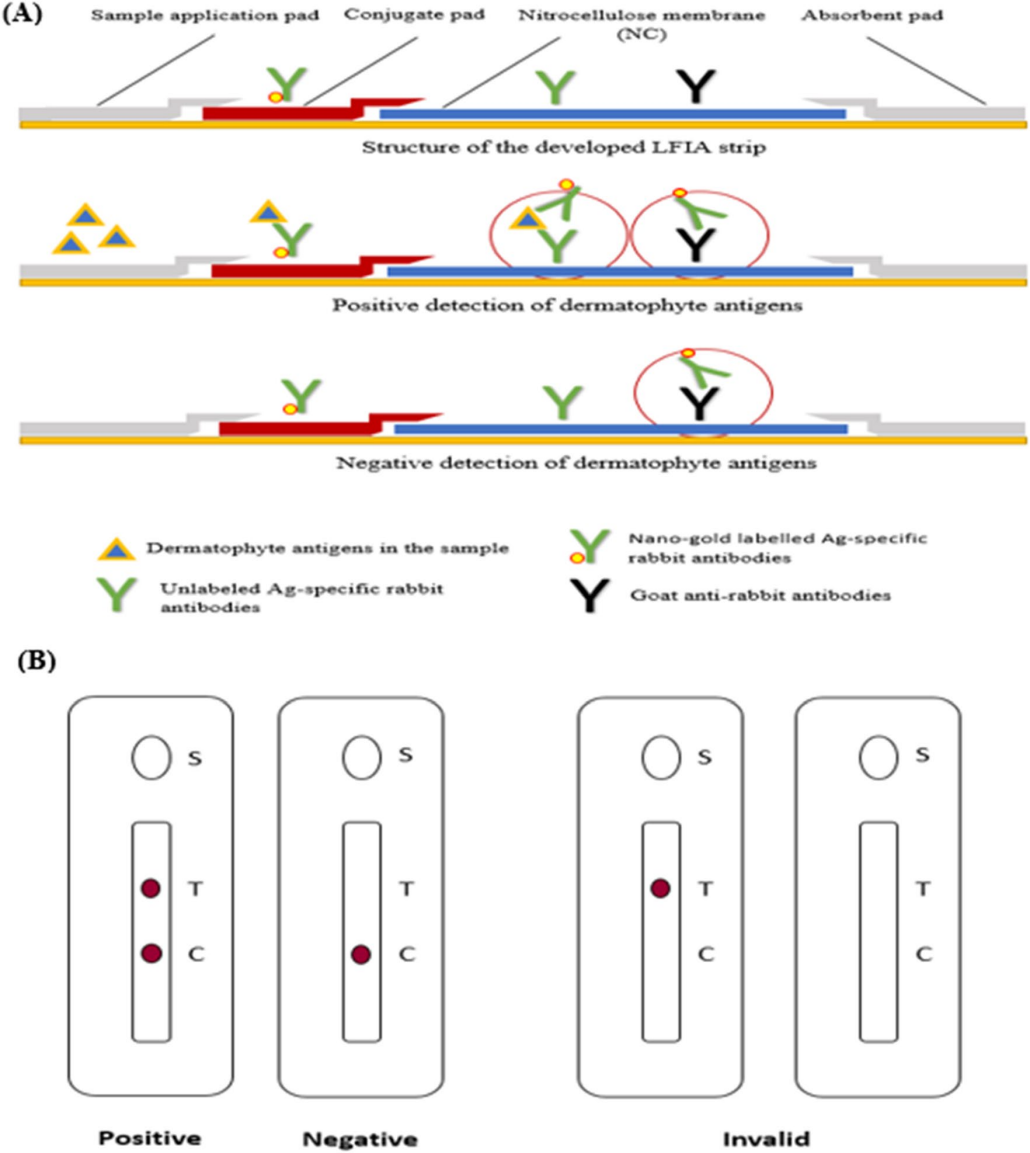
(**A**) Diagrammatic illustration of the working principle of the newly developed kit. (**B**) Illustration of the valid (positive/negative) and the invalid visible outcome results.

**Figure 2:**
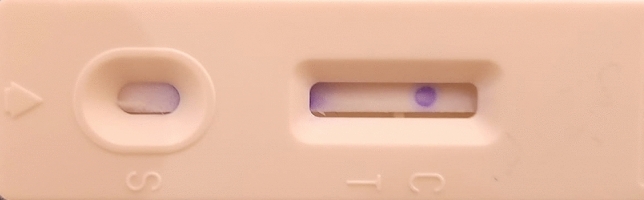
Demonstration of the negative reaction appearance in the newly developed kit.

**Figure 3:**
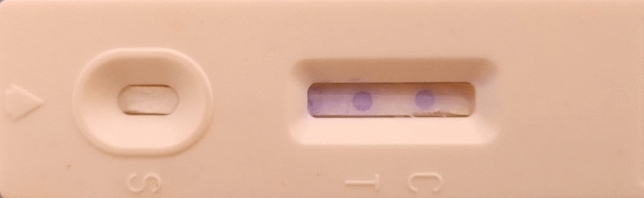
Demonstration of the positive reaction appearance in the newly developed kit.

**Figure 4:**
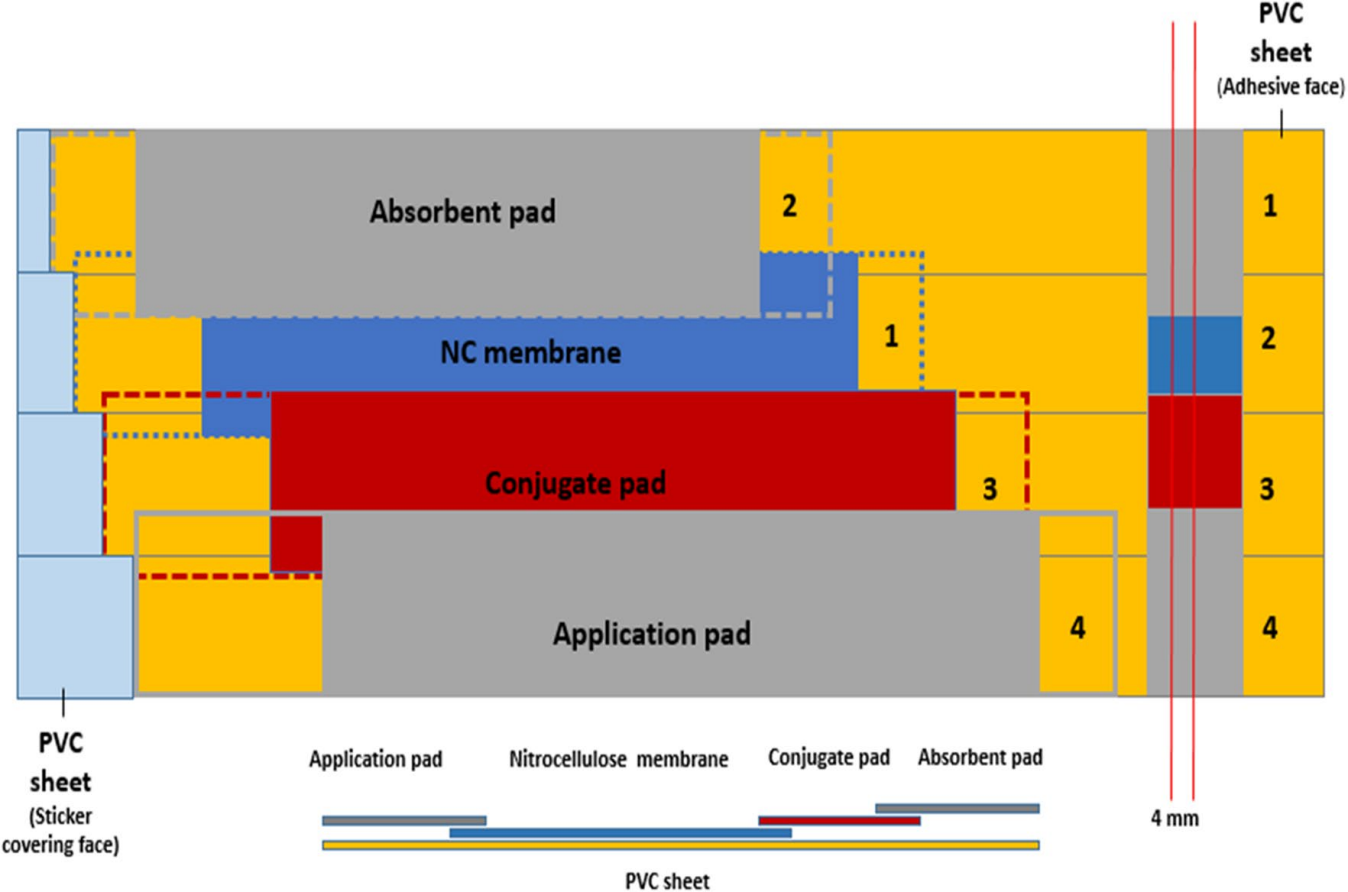
Diagrammatic illustration of the main steps to build up the laminated lateral flow solid phase with a diagrammatic illustration of the building structures of each single lateral flow strip.

Direct antigen–antibody interaction was demonstrated to form an Antigen–Antibody-Au NPs-pAbs complex for the detection of dermatophyte antigens presence in hair, nail, and skin scrap samples. An appropriate amount of sample was added to the antigen extraction solution; the sample then was applied in the sample application window in the LFIA cartilage. The sample moved forward along the strip under the capillary effect. If the sample contains target dermatophyte antigens, the antigens will bind to the labeled rabbit anti-dermatophytes IgG-pAbs present in the conjugate pad of the cassette, and the complex would migrate to the membrane-bound unlabeled anti-dermatophytes IgG on the test spot turning it to ruby red, showed that the sample was positive. On the other hand, the unbound conjugates continued to flow forward and bound to unlabeled goat anti-rabbit pAbs (INVITOGEN-THERMO-FISHER) on the control spot, turning it ruby red also. If the sample does not contain the target dermatophyte antigens, only the control spot will turn ruby red. Therefore, the LFIAs are positive if two ruby red spots appeared on both test and control spots while it was negative if only one ruby red dot appeared at the control spot. Invalid strips showed no ruby red dots either in the test and control spots or showed only one ruby red dot in the test spot as illustrated in Figs. 1, 2, 3, and 4.

### Post-kit development stage (evaluation stage)

#### Specificity and stability of the newly developed LFIA strips

The specificity of the newly developed LFIAs was tested by both laboratory-based antigens Table 1 as well as by employing cutaneous samples including samples of clinically diagnosed cases of dermatophytosis, with alopecia, itching, and skin crusting. Each sample was tested in duplicates at two weeks without any changes, demonstrating the excellent stability of the LFIA strips produced. Further prospective studies are needed for further investigation of cross-reactivity with other cutaneous affection causes.

**Table 1:** Illustration of the validating testing outcome, of different keratinophilic dermatophytes and non-dermatophytes antigens, by both the newly developed kit and the agar gel precipitation test.

			Multispecies Dermato-Kit	AGPT using multispecies pAbs cocktail in the central well
Dermatophytes group	*Microsporum canis*	Soluble Ag	Positive	Positive
		Insoluble Ag	Positive	Negative
	*Trichophyton verrucosum*	Soluble Ag	Positive	Positive
		Insoluble Ag	Positive	Negative
	*Trichophyton mentagrophytes*	Soluble Ag	Positive	Positive
		Insoluble Ag	Positive	Negative
	*Trichophyton equinum*	Soluble Ag	Positive	Positive
		Insoluble Ag	Positive	Negative
	*Microsporum gypsum*	Soluble Ag	Positive	Positive
		Insoluble Ag	Positive	Negative
Non-dermatophyte groups	**Yeasts**			
	*Candida* species Ag mixture		Negative	Negative
	*Malassezia* species Ag mixture		Negative	Negative
	*Rhodotorula* species Ag mixture		Negative	Negative
	**Molds**			
	Hyaline fungi	*Aspergillus* species soluble Ag mixture	Negative	Negative
		*Penicillium* species soluble Ag mixture	Negative	Negative
		*Fusarium* species soluble Ag mixture	Negative	Negative
	Dematiaceous soluble Ag		Negative	Negative

Soluble Ag; is the submerged growth derived Ag, while insoluble Ag; is the superficial mycelial growth derived Ag.

#### Testing of the laboratory-prepared antigens and the clinical dermatological samples by the LFIA strips

Regarding the laboratory-prepared antigens testing, it was remarkably noted that all non-dermatophyte molds are negative, which indicates that the specificity of the prepared polyclonal antibody is very high.

Clinical practicability of the LFIAs was evaluated through dermatological sample testing to detect cutaneous samples collected from 100 skin affections suffered cases a few days (1–3 days) after their clinical diagnosis, where culture-negative confirmed cutaneous samples were used as the negative control. All KOH direct microscopic examinations, standard cultures, and LFIA results are summarized in Tables 2, 3 and 4.

**Table 2:** Comparing LFIA and microscopic slide results.

	Results	Direct microscopic examination (DME)	
		Positive	Negative
LFIA	Positive	6	9
	Negative	3	82

**Table 3:** Comparing culture and LFIA results.

	Results	LFIA	
		Positive	Negative
Culture	Positive	14	6
	Negative	1	79

**Table 4:** Comparing microscopic slide and culture results.

	Results	Culture	
		Positive	Negative
DME	Positive	5	4
	Negative	15	76

The specificity and sensitivity of the newly developed kit were calculated as follows;

$${\text{Specificity}} = {\text{T}} - /\left( {\left( {{\text{T}} - } \right) \, + \, \left( {{\text{F}} + } \right)} \right) \, \times { 1}00\% ,$$

$${\text{Sensitivity}} = {\text{T}} + /\left( {\left( {{\text{T}} + } \right) \, + \, \left( {{\text{F}} - } \right)} \right) \, \times { 1}00\% .$$

The LFIAs gave a specificity of 98% and a sensitivity of 76.9%. Cohen’s kappa statistic was calculated to measure the level of agreement between the newly developed kit and the standard culture as follows;

$${\text{Cohen's kappa coefficient }}({\text{k}}) \, = \, \left( {{\text{p}}_{{\text{o}}} {-}{\text{ p}}_{{\text{e}}} } \right)/\left( {1 \, {-}{\text{ p}}_{{\text{e}}} } \right),$$

where (p_o_) is the relative observed agreement among raters and (p_e_) is the hypothetical probability of chance agreement.

The result was 0.44 interpreted as “moderate agreement” with the direct microscopy representing an observable dissociation between the two raters which also can be accounted for the new kit due to the low specificity, sensitivity, and agreement of the direct microscopy with gold standard culturing. On the other hand, the result was 0.76 interpreted as a “substantial agreement” with the standard culture. Although the LFIAs have many advantages, after all, LFIA is usually only used to acquire a preliminary diagnosis. With the evaluation of the performance, the current study is helpful to explore future diagnostic applications in the LFIA field. A possible improvement strategy focusing on identifying new signal amplification strategies and quantification systems warrants further study. The lateral flow-based techniques are considered a promising competitor to both conventional laboratory-based diagnostic methods and advanced molecular-based diagnostic techniques. Enhancing the lateral flows’ diagnostic accuracy, sensitivity, and specificity is the recent challenge and prospect for dermatophytosis diagnosis.

To conclude, the conducted study described the development, optimization, and validation of a new point-of-care (POC) and field test based on the specific interactions between dermatophyte antigens and dermatophyte antigens-specific antibodies. Achieving an accurate diagnosis by detecting the dermatophyte antigens in hair, nail, and skin scrap samples from dermatologically affected animals as a substitution to the most commonly used and rapid, however imperfect direct wet prepared slides and the time-consuming expensive specific culturing. The newly developed lateral flow immunochromatographic assays (LFIAs) can potentially provide a preliminary and competitive test result for clinicians to make the appropriate diagnosis and provide initial proper treatment to affected cases along with alternative testing methods and clinical findings. Moreover, the applicability of the new LFIAs is faster and easier than other methods such as direct wet mounts, implementation of isolation, and molecular techniques, especially in most medical facilities as well as remote countryside and low-income countries or regions. With the widespread nature of dermatophyte infections, it becomes an essential demand to achieve an accurate, rapid, specific, and sensitive diagnosis for dermatophytosis. On this occasion, the current serological LFIAs are supposed to be a more efficient and cost-effective solution to deal with dermatophytes’ affections through three simple steps and within 5–7 min from the sample collection till reading the result. Also, it is worth being clarified that through 100 clinical sample testing, the newly developed kit showed no actual limit of detection (LoD) related to the type and nature of the collected sample or to the clinical stage of the diseases or others. However, prospectively, further and a larger number of sample testing is required to completely cover the LoD-related point.

## Materials and methods

### Materials

#### Pre-kit development stage

Sabouraud’s dextrose agar (SDA)-Sabouraud’s dextrose broth (SDB)-Chloramphenicol-Cycloheximide-Phosphate-buffered saline (PBS)-Complete Freund’s adjuvant (CFA)-Incomplete Freund’s adjuvant (IFA)-Formalin-Caprylic acid-12,000–14,000 molecular weight cut-off (MOWC) dialysis bags-Ultra-pure water-Agarose-Distilled water.

#### Kit development stage

Ultra-pure water, 0.2% HauCl4.3H2O (Gold III chloride trihydrate)—1% Sodium citrated buffer-Tris-Sodium azide-Sucrose-Bovine serum albumin (BSA)-Sample pad roll-Conjugate pad roll-Nitrocellulose membrane roll-Absorbent pad roll-Adhesive polyvinyl chloride (PVC) sheets**-**Unlabeled goat anti-rabbit antibodies-Glycerin-Propylene glycol-Sodium dodecyl sulfate (SDS)-Dimethyl sulfoxide (DMSO)-Diazolidinyl urea-Distilled water-Lateral flow cartilages.

#### Post-kit development stage

Potassium hydroxide (KOH) 10%-Dimethyl sulfoxide (DMSO)-Glycerine-Lactophenol cotton blue (LPCB)-Sabouraud’s dextrose agar (SDA)-Nicotinic acid-Inositol-Thiamine-Chloramphenicol-Cycloheximide.

### Methodology

#### Pre-kit development stage (preliminary stage)

##### Fungal strains obtaining

Five hot local dermatophytes strains belong to the following species: *Microsporum canis* (*M. canis*), *Microsporum gypseum* (*M. gypseum*), *Trichophyton equinum* (*T. equinum*), *Trichophyton verrucosum* (*T. verrucosum*), and *Trichophyton mentagrophytes* (*T. mentagrophytes*), were used during the current study. The used strains were isolated, identified, and selected through a previous preliminary cross-sectional study on dermatological affections of companion animals caused by dermatophytes and other keratinophilic fungi in the Greater Cairo Area, Egypt, which has been conducted by the same scientific research team^23^. Those strains have been used for the preparation of antigens that were used for the immunization of animals during the lateral flow kit development stages, and also were used as a positive control during the newly developed kit validation stage.

##### Selection of dermatophyte strains

From the isolated dermatophyte species, the most suitable strains for vaccine production were selected according to the criteria reported by Ref.^24^. According to these criteria, we found that dermatophyte vaccinal strain should be:

1. Local strain.
2. Isolated from badly infected cases.
3. Rapid grower in vitro.
4. Intensive spore formers, where the dermatophyte microconidia are considered as an important character for the selection of suitable vaccinal strain. This is because microconidia have been identified to be the carrier of immunogenicity.
5. High growth intensity that allows the production of a large number of fungal antigens.

##### Preparation of dermatophyte antigens

Mycelial matt antigens were prepared for each dermatophyte of the five species involved in the current study as follows; The isolated, identified, and selected strains have been cultured separately on non-supplemented Sabouraud’s dextrose broth, after 21 days, harvesting and separating of the superficial floating mycelial matt from the submerged soluble fungal structures using sterile gauze^25^. Each harvested mycelial matt was homogenized using a homogenizer at 160 rpm for 5 min, then washed with sterile phosphate-buffered saline (PBS) three times, each time PBS suspension, centrifugation at 5000 rpm for 5 min, and discard of the supernatant were performed. Finally, each mycelial antigen was suspended in PBS to a concentration of 4%.

##### Preparation of emulsions used for animal immunization

The immunization emulsion for dermatophytes mycelial antigen was prepared as follows; the priming immunization emulsion consisted of equal volumes of complete Freund’s adjuvant (CFA, SIGMA-ALDRICH) and inactivated previously prepared 4% mycelial antigens, inactivation was performed using 0.5% formalin (SIGMA-ALDRICH), mixed adequately using two syringes and connector for 20 min till reached a milky white viscous creamy emulsion stable after overnight testing at refrigerator temperature (6 °C). The boostering immunization emulsion consisted of equal volumes of incomplete Freund’s adjuvant (IFA, SIGMA-ALDRICH) and living previously prepared 4% mycelial antigens, mixed adequately using two syringes and connector for 20 min till reached a milky, white, viscous, and creamy emulsion with stable formulation after overnight testing at refrigerator temperature (6 °C)^26^. Both types of immunization emulsions have been prepared and injected under aseptic techniques.

#### Lab animal selection and immunization protocol design

##### The selected lab animal

Two white male New-Zealand rabbits weighing 2 kg were designed to be used as the bio-factory for dermatophytes pAbs production. As using a fully mature animal will ensure a completely functioning immune system which is the cornerstone of antibody production. Also, rabbits were the chosen lab animal from the commonly used lab animal list used for antibody production as it is characterized by their small size, high affinity, relatively long life span, easy blood obtaining, strong immune response, and inexpensive housing^27^. A number of two animals were injected with the same dermatophytes Ag to enlarge the obtained blood samples carrying antibodies for each Ag, also to take in advance the unexpected accidental death of one of the two animals before the end of the injection protocol.

##### The design of the animal immunization protocol

Based on the standardization protocols of animal immunization in association with the regular monitoring of the production of antibodies using Ouchterlony’s test the conducted animal immunization protocol was designed and performed^28^ (Table 5).

**Table 5:** Illustration of a detailed animal immunization schedule.

Day 0	A blood sample was collected from each animal involved in the conducted study	
Day 1	0.5 ml/kg of the priming emulsion	The whole dose is injected intradermally (I/D) and divided into different 10 sites
Day 14	1 ml/kg of the boostering emulsion	The whole dose is injected subcutaneously (S/C) and divided into 5 different sites
Day 28	1 ml/kg of the boostering emulsion	The whole dose is injected S/C and divided into 5 different sites
**Ear vein blood samples collection and Agar gel precipitation tests were performed**		
Day 38	1 ml/kg of the boostering emulsion	The whole dose is injected S/C and divided into 5 different sites
**Ear vein blood samples collection and Agar gel precipitation tests were performed**		
Day 48	1 ml/kg of the boostering emulsion	The whole dose is injected S/C and divided into 5 different sites
**Ear vein blood samples collection and Agar gel precipitation tests were performed**		
Day 55	1 ml/kg of the boostering emulsion	Half of the dose S/C is divided into 2 different sites and the other half of I/M is divided into 2 different sites
**Ear vein blood samples collection and Agar gel precipitation tests were performed**		
Day 62	1 ml/kg of the boostering emulsion	Half of the dose is injected S/C and divided into 2 different sites and the other half is injected intramuscularly (I/M) and divided into 2 different sites
**Ear vein blood samples collection and Agar gel precipitation tests were performed**		
Day 79	1 ml/kg of the boostering emulsion	Half of the dose is injected S/C and divided into 2 different sites and the other half is injected I/M and divided into 2 different sites
**Ear vein blood samples collection and Agar gel precipitation tests were performed**		
Day 90	Final blood collection	

#### Separation and purification of polyclonal antibodies (pAbs)

The separately collected blood samples were kept for 1 h undisturbed in a slightly oblique position at room temperature (25 °C), followed by another hour in the refrigerator (6 °C). Then 25 ml of the blood was centrifuged for 30 min at 10,000×*g*, the supernatant was collected, and the pellet was discarded. The obtained serum was placed on a magnetic stirrer, then dropwise of caprylic acid, 2.02 ml/25 ml rabbit serum was dropped slowly while stirring at 25 °C for 30 min. The mixtures were centrifuged at 10,000×*g* for 20 min, the supernatants were collected, and the pellets were discarded. The collected supernatants were dialyzed separately against PBS at 4 °C overnight using 12,000–14,000 molecular weight cut-off (MOWC) dialysis bags (SIGMA-ALDRICH) with three buffer changes, finally, the concentration of immunoglobulins was obtained by measuring both the total protein and the albumin concentrations of each obtained purified antibodies sample from the dialysis bags^29^. The obtained immunoglobulins concentrations ranged from 1.1 to 1.4 g/dl. A 1 mg/ml concentration was then obtained using ultrapure water dilution. Furtherly, half the volume of the separately obtained dermatophyte species-specific polyclonal antibodies were pooled together in equal ratios to form dermatophyte multispecies-specific polyclonal antibodies (pAbs).

#### Agar gel precipitation test optimization for evaluating the separated pAbs

Agar gel plates were prepared as follows; 3 mm agar gel thickness and 0.6% concentration were achieved by dissolving 0.6 g agarose in 100 ml PBS, then dissolved by boiling for 2 min and pouring 4 ml from the preparation in 5 cm plates, then left to solidify^30^. The agar gel test was used in several situations during the current study; First, to monitor the antibody production during the designed immunization protocol, second, to evaluate the consequence of the pAbs purification steps, and third, to study the relationship between the produced species-specific pAbs and the prepared immunizing antigens, which derived from different dermatophyte species and the relationship between the produced dermatophyte multi-specific pAbs and other non-dermatophyte keratinophilic fungal antigens.

### Kit development stage (developmental and standardization stage)

#### Preparation of colloidal gold (CG) nanoparticles (NPs)

50 ml of ultrapure water was placed on a hot plate till boiling (100 °C), then vigorous magnetic stirring was started followed by the addition of 0.5 ml of 0.2% HauCl4·3H2O (Gold III chloride trihydrate) (SIGMA-ALDRICH). During the boiling of the mixture with vigorous stirring, 1 ml of 1% (w/v) sodium citrated buffer was added quickly. The color of the mixture began as a colorless solution changed to black then ruby red color (after approximately 2 min), then kept on stirring and at the boiling temperature (100 °C) for another 10 min^31–33^. The prepared nanoparticles were scanned at a range of 400–600 nm using a UV–Vis spectrophotometer and Transmissible electron microscope (TEM) imaging to determine their diameter.

#### Conjugation of the purified rabbit polyclonal antibodies with the colloidal gold nanoparticles (Au NPs-pAbs coupled bio-conjugate)

Half milliliter of purified multi-species rabbit pAbs (1 mg/ml) was added to 50 ml of colloidal gold nanoparticles. The solution was gently mixed for 10 min. Then centrifuged for 10 min at 10,000×*g*, discarding the supernatant and keeping the pellet. The conjugated pellet was diluted in 1 ml conjugation dilution buffer, which was prepared as follows; 20 mM Tris, 0.02%(w/v) sodium azide, 3% (w/v) sucrose, and 1% (w/v) bovine serum albumin (BSA)) and stored in a refrigerator (6 °C)^20–22^.

#### An overall newly developed kit fabrication and formulation procedures

##### Preparation of the lateral flow solid phase (multi-laminated membranes strip)

The newly developed LFA solid phase was prepared as follows; Sample pad roll (AHLSTROM): pre-treated glass fiber with sample pad treatment solution pH 7.4 (ultra-pure water, 2% (w/v) Triton X-100, 3.81% (w/v) Borax, 1% (w/v) Polyvinylpyrrolidone (PVP), 0.5%(w/v) sodium cholate, 0.15% (w/v) sodium dodecyl sulfate (SDS), 0.1% (w/v) casein sodium salt, 0.02% (w/v) sodium azide, then dried for 1 h at 37 °C. Conjugation pad roll (AHLSTROM): pre-treated glass fiber with conjugation treatment solution pH 7.4 (2.5% (w/v) sucrose, 20 mM PBS, 0.3% (w/v) PVP, 2% (w/v) BSA, 1% (w/v) Triton X-100 and 0.02% (w/v) sodium azide). Nitrocellulose membrane (NC) roll (AHLSTROM): Microporous nitrocellulose membranes are used as the carrying matrix upon which Ag–Ab immune-complexes are formed and visualized. Absorbent (wick) pad roll (AHLSTROM): a cotton fiber pad roll, whose function is to collect the liquid processed through the strip by capillary action. Adhesive Polyvinyl chloride (PVC) sheets (AHLSTROM): A four-row laminated adhesive membrane as the carrier base of the rest components of the lateral flow strip, starting with removing the sticker from the second row and attaching the nitrocellulose membrane, followed by removing the sticker from the first row and attaching the absorbent pad, the third step was removing the sticker from the third row and attaching the conjugate pad, finally removing the sticker from the fourth row and attaching the sample pad which is diagrammatically illustrated in Fig. 4.

##### Loading of the different bio-reactants on the prepared lateral flow solid phase

To yield clear and accurate results, coating anti-dermatophytes pAbs and goat anti-rabbit IgG pAbs were optimized. The red on the C line was visible when the concentration of goat anti-rabbit IgG was more than 0.3 mg/ml and became clearest as the coating concentration increased to 0.5 mg/ml. As for the T line, the color spot and uniform distribution were observed when the coating concentration was more than 0.5 mg/ml. To meet the requirements of the test strip observation, 1 mg/ml of anti-dermatophytes pAbs and 0.5 mg/ml of the polyclonal goat anti-rabbit IgG antibody (pH 7.4) were chosen as the optimal concentration^34,35^. The antibody printer (ISOFLOW) was used to load the 0.5 μl of unlabeled goat anti-rabbit antibodies in the form of dots on the control spots, while the test spots were loaded as follows: Single-plex edition: This is a single test spot dotted edition, which was developed in 6 versions; 1 dermatophyte multi-species version that was dotted with 1 μl of dermatophyte multi-species pAbs at the test spot and 5 dermatophytes species-specific versions dotted with 1 μl of dermatophyte species-specific pAbs at the test spot. Multiplex edition: This is a multi-test spot dotted edition.

The space between the test spot and the control spot was adjusted to be 0.5 cm in the single-plex edition. The conjugate pad was loaded by impregnation in the conjugate solution achieving 5 μl conjugate on each cut conjugate pad of each strip.

##### Cutting of the test strips and packaging in lateral flow cartilage and the kit assembly

**Figure 5:**
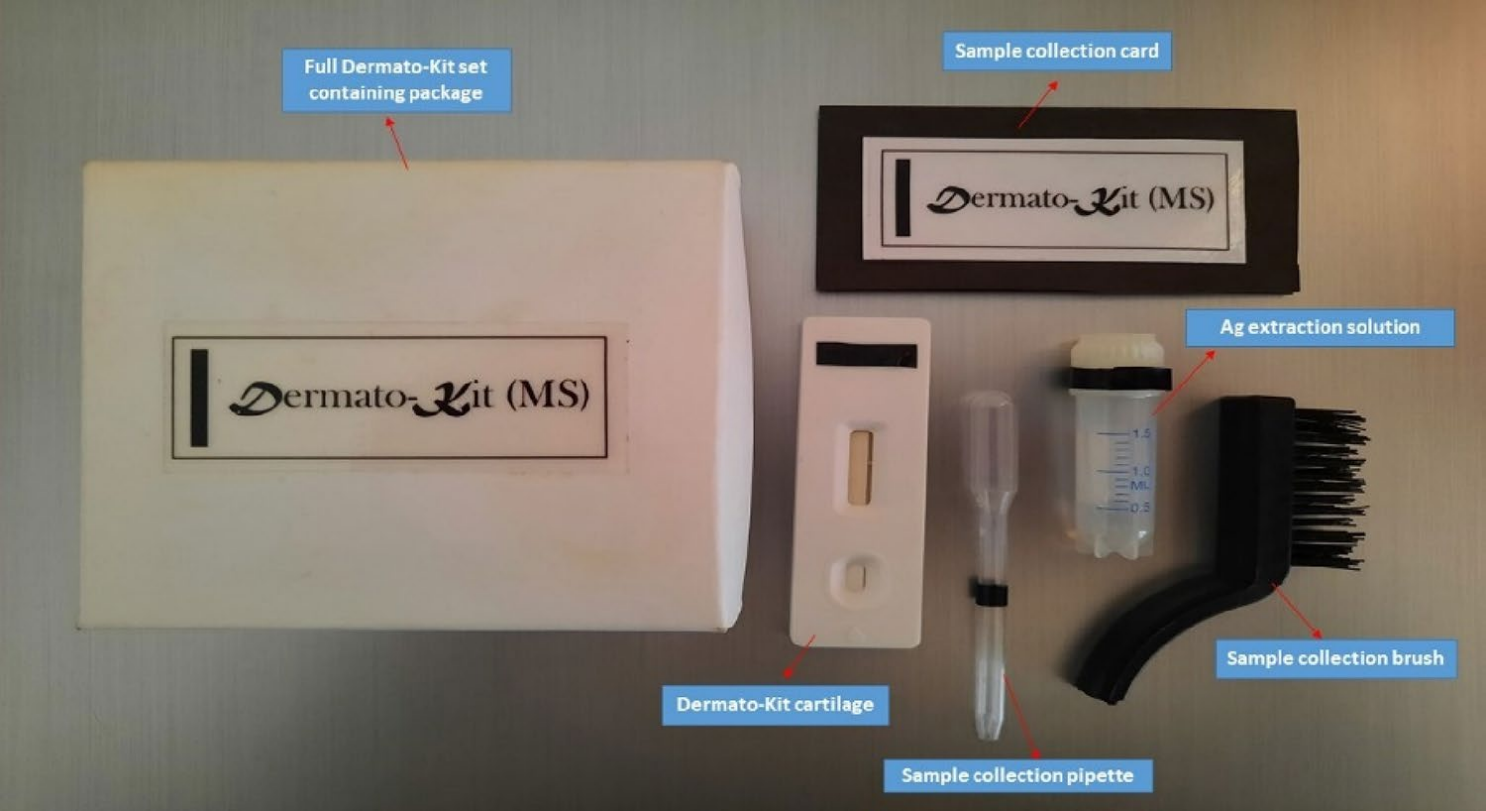
The newly developed Dermato-kit components.

The loaded adhered to the PVC card developed lateral flow sheets, automatically were cut by an automatic Guillotine cutter achieving 0.4 cm width per strip, then due to the research level production quantity, manual assembly, and packaging of the strips into the lateral flow cartilage has occurred. The used cartilage is characterized by (S) letter labeled sample application window and (T and C) letter labeled window marking the test and control spots on the strip. The kit was assembled as a full set containing, the LFIA cartilage/cassette, scrapping brush, sample collection card, antigen extraction solution, and sample application pipette Fig. 5.

#### Preparation of antigen extraction solution

Based on several laboratory-based trials and previously published literature^25,36–40^ this solution was formulated and prepared as follows: 5 ml glycerin, 1 ml propylene glycol, 1 g sodium dodecyl sulfate (SDS), 1 g dimethyl sulfoxide (DMSO), and 0.2 g Diazolidinyl urea were stirred and dissolved in 94 ml distilled water.

### Post-kit development stage (evaluation stage)

#### Pre-field challenge laboratory testing for different keratinophilic dermatophytes and non-dermatophytes fungi

A group of antigens representing most of the keratinophilic dermatophytes and non-dermatophytes fungi has been tested and compared with the AGPT, as a laboratory-based evaluatory step of the prepared dermatophytes pAbs specificity incorporated in the newly developed kit.

#### Collection of the samples used during the developed lateral flow testing and challenge with the wet mounts and cultures

A total of 100 samples (Skin scraps, scales, and hair) were collected from a wide range of companion animal species; Dogs, cats, horses, donkeys, cows, buffalos, rabbits, rats, and guinea pigs. All samples have been collected from animals suffering from dermatological affections and suspected by their clinician it would be a dermatophyte infection. All samples have been collected nearly by the same standardized sampling protocol by the hands of expert veterinarians and submitted either to LEPTOVET (an ISO 9001 accredited and licensed veterinary laboratory in Egypt) or to the mycology unit at the department of microbiology, faculty of veterinary medicine, Cairo University. These samples were directly tested through wet mounts and then confirmed as positive active dermatophytosis cases or negative cases by the gold standard selective dermatophytes culturing techniques^23^.

#### Direct microscopic examination of the collected samples

Few hairs, and/or skin scraping were placed on a sterile slide. A few drops of potassium hydroxide (KOH) 10% with dimethyl sulfoxide (DMSO, SERVA) and glycerin (SERVA) were added to the sample (DMSO enhances KOH 10% clarifying ability and glycerin as a source of humidity-reducing the required incubation time)^41,42^. The slide was covered with a coverslip, heated gently, left in a humid chamber for 30 min, and examined using low and high dry power lenses of a bright-field microscope (Olympus CX31 microscope with camera model C-7070).

#### Culturing of the collected samples

A total of 100 samples were examined by culturing on Sabouraud’s dextrose agar (SDA, OXOID) supplemented with 0.5 g/l chloramphenicol (OXOID) and 0.5 g/l cycloheximide (OXOID). Each sample was inoculated in 3 sets of supplemented SDA slopes and each set consisted of 3 slopes. The first set was only supplemented with the basic selective supplements (Chloramphenicol and cycloheximide) the second set was extra supplemented with specific supplements (thiamine (SERVA) and inositol (SERVA)) and the third set extra supplemented with different specific supplements, (Nicotinic acid (SERVA)). The first and third sets were incubated at 25 °C and observed for up to 21 days while the second set was incubated at 30 °C and observed for up to 30 days^23,41^.

#### The newly developed kit testing procedures

Three brushing from the active peripheral border of the suspected lesion using the scrapping brush. The sample was collected in a black collection card to assess the amount of the collected sample (Fig. 5). Then the sample was added to a 0.5 ml extraction solution with gentle mixing for 5 min at room temperature (25 °C). 100 μl (3 drops) of the extracted antigen was applied to the sample application window using the application pipette. The presence of dermatophyte antigens was detected by the appearance of a specific color tracer of Au NPs Supplementary 2.

### Approval for animal experiments

The current conducted study is reported in accordance with (Animal Research: Reporting of In-Vivo Experiments-ARRIVE) guidelines. The guidelines of the (Institutional Animal Care and Use Committee-IACUC of the faculty of veterinary medicine, Cairo University) were completely followed during any procedures involving animal use through the current conducted study. This study is approved by the ethical committee of the institutional animal care and use committee (IACUC) of Veterinary-Cairo University-2305-2022478.

No anesthesia or euthanasia protocols were used with the animal involved during this study as all animal-dependent methodological procedures were considered as no to low pain-causing procedures that ethically can be done on a conscious alive animal.

## Supplementary Information

Supplementary Information 1.

Supplementary Information 2.

## Data Availability

The datasets used and/or analyzed related to the animal cases tested during the current study are available from the corresponding author upon reasonable request.
