# Supplementary material for: Development, preparation, and evaluation of a novel dotted lateral flow immunochromatographic kit for rapid diagnosis of dermatophytosis
Source: Sci Rep. 2023 Jan 5;13:248. doi: 10.1038/s41598-023-27443-4 (PMC9816107; doi:10.1038/s41598-023-27443-4)
Supplement: Supplementary file 1 — Supplementary Information 1. [file 41598_2023_27443_MOESM1_ESM.docx]

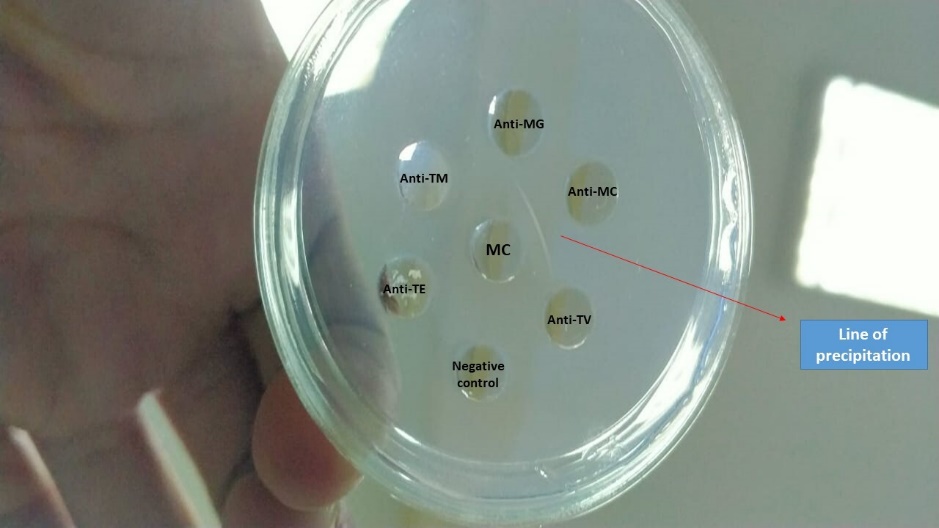


**A**

**Supplementary figure 1 (A)**: illustrate the line of precipitation that appears only, between *Microsporum canis* antigen and anti-*Microsporum canis* produced antibodies. MC; *Microsporum canis*, MG; *Microsporum gypseum*, TV; *Trichophyton verrucosum*, TM; *Trichophyton mentagrophytes*, TE; *Trichophyton equinum*, and saline were used as a negative control.


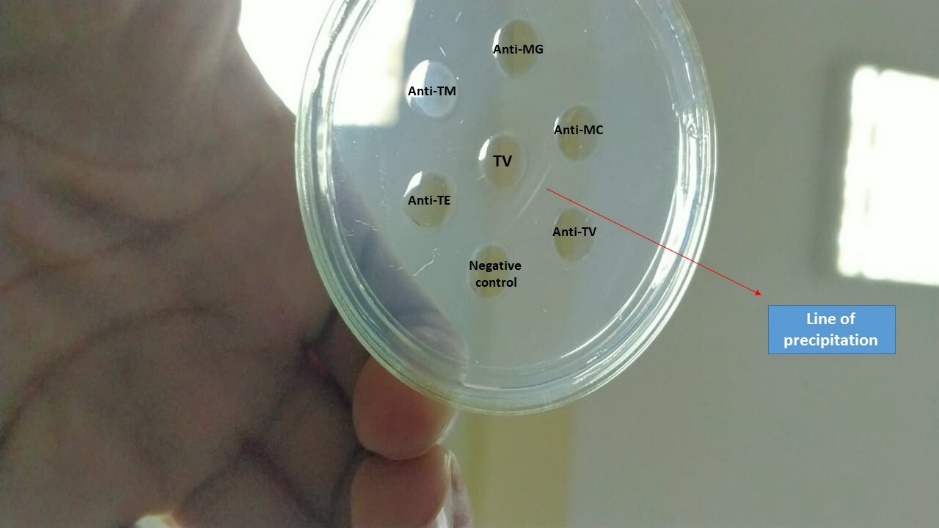


**B**

**Supplementary figure 1 (B)**: illustrate the line of precipitation that appears only, between *Trichophyton verrucosum* antigen and anti-*Trichophyton verrucosum* produced antibodies. MC; *Microsporum canis*, MG; *Microsporum gypseum*, TV; *Trichophyton verrucosum*, TM; *Trichophyton mentagrophytes*, TE; *Trichophyton equinum*, and saline were used as a negative control.


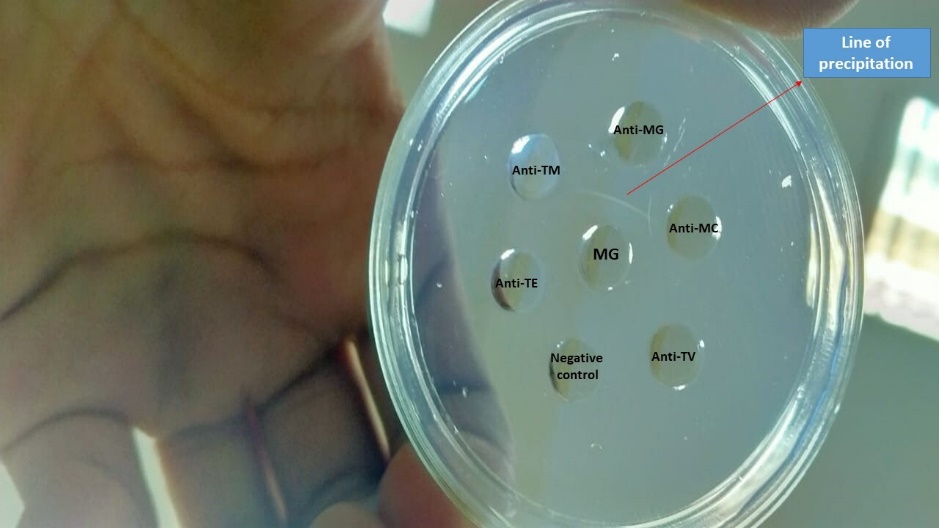


**C**

**Supplementary figure 1 (C)**: illustrate the line of precipitation that appears only, between *Microsporum gypseum* antigen and anti- *Microsporum gypseum* produced antibodies. MC; *Microsporum canis*, MG; *Microsporum gypseum*, TV; *Trichophyton verrucosum*, TM; *Trichophyton mentagrophytes*, TE; *Trichophyton equinum*, and saline were used as a negative control.


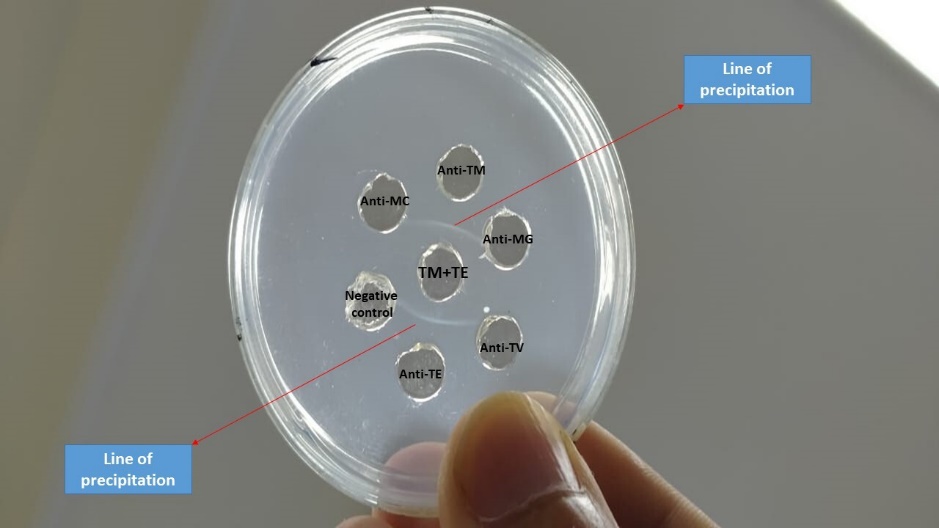


**D**

**Supplementary figure 1 (D)**: illustrate the lines of precipitation that appear only, between *Trichophyton mentagrophytes* and *Trichophyton equinum* mixed antigens and both anti*-Trichophyton mentagrophytes* and anti- *Trichophyton equinum* produced antibodies. MC; *Microsporum canis*, MG; *Microsporum gypseum*, TV; *Trichophyton verrucosum*, TM; *Trichophyton mentagrophytes*, TE; *Trichophyton equinum*, and saline were used as a negative control.


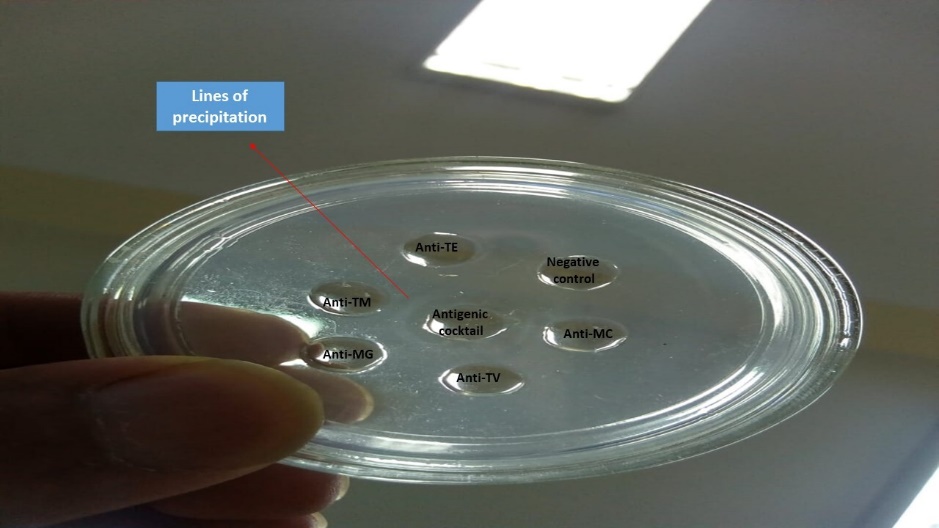


**E**

**Supplementary figure 1 (E)**: illustrate the lines of precipitation that appear, between dermatophytes antigenic cocktail (*Microsporum canis, Microsporum gypseum, Trichophyton verrucosum*, *Trichophyton mentagrophytes,* and *Trichophyton equinum* mixed antigens) and species-specific anti-dermatophytes produced antibodies. MC; *Microsporum canis*, MG; *Microsporum gypseum*, TV; *Trichophyton verrucosum*, TM; *Trichophyton mentagrophytes*, TE; *Trichophyton equinum*, and saline were used as a negative control.


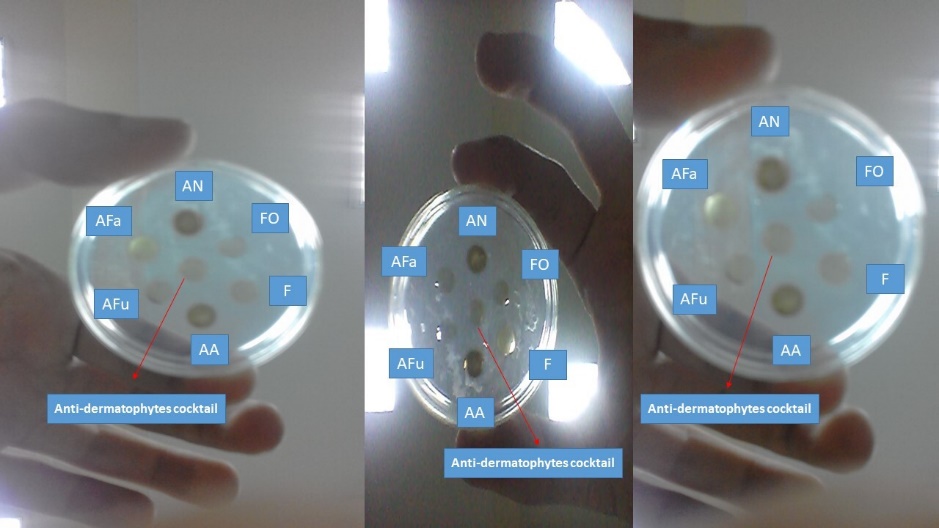


**F**

**Supplementary figure 1 (F)**: illustrate the absence of any precipitation lines between anti-dermatophytes cocktail produced antibodies and different non-dermatophytes keratinolytic antigens. AFa; *Aspergillus flavus*, AFu*; Aspergillus fumigatus*, AA; *Alternaria alternate*, F; *Fusarium chlamydosporum*, FO; *Fusarium oxysporum*, AN; *Aspergillus niger*.
