# Supplementary material for: Development, preparation, and evaluation of a novel dotted lateral flow immunochromatographic kit for rapid diagnosis of dermatophytosis
Source: Sci Rep. 2023 Jan 5;13:248. doi: 10.1038/s41598-023-27443-4 (PMC9816107; doi:10.1038/s41598-023-27443-4)
Supplement: Supplementary file 2 — Supplementary Information 2. [file 41598_2023_27443_MOESM2_ESM.docx]

Supplementary material for this article is available online at <https://www.youtube.com/playlist?list=PL1uGfMXH_u0uNsBrNaFlaD82OcXouiq10>.

A series of videos illustrate all the applicability steps and the outcome results are available online at the attached link
